# Can mobile phone surveys accurately measure the coverage of maternal healthcare services? Results from the Rapid Mortality Mobile Phone Survey (RaMMPS) study in Mozambique

**DOI:** 10.1093/oodh/oqag011

**Published:** 2026-06-05

**Authors:** Helen Kuo, Almamy Malick Kante, Cremildo Manhica, Celso Monjane, Aveika Akum, Fred Van Dyk, Nordino Machava, Azarias Mulungo, Ivalda Macicame, Agbessi Amouzou

**Affiliations:** Department of International Health, Johns Hopkins Bloomberg School of Public Health, 615 N. Wolfe Street, Baltimore, MD 21205, United States; Department of International Health, Johns Hopkins Bloomberg School of Public Health, 615 N. Wolfe Street, Baltimore, MD 21205, United States; Centro de Investigação e Treino em Saúde da Polana Caniço, Instituto Nacional de Saude, EN1, Bairro da Villa Parcela N#3943, Distrito de Marracuene, Provincia de Maputo, Mozambique; Centro de Investigação e Treino em Saúde da Polana Caniço, Instituto Nacional de Saude, EN1, Bairro da Villa Parcela N#3943, Distrito de Marracuene, Provincia de Maputo, Mozambique; Department of International Health, Johns Hopkins Bloomberg School of Public Health, 615 N. Wolfe Street, Baltimore, MD 21205, United States; Department of International Health, Johns Hopkins Bloomberg School of Public Health, 615 N. Wolfe Street, Baltimore, MD 21205, United States; Centro de Investigação e Treino em Saúde da Polana Caniço, Instituto Nacional de Saude, EN1, Bairro da Villa Parcela N#3943, Distrito de Marracuene, Provincia de Maputo, Mozambique; Centro de Investigação e Treino em Saúde da Polana Caniço, Instituto Nacional de Saude, EN1, Bairro da Villa Parcela N#3943, Distrito de Marracuene, Provincia de Maputo, Mozambique; Centro de Investigação e Treino em Saúde da Polana Caniço, Instituto Nacional de Saude, EN1, Bairro da Villa Parcela N#3943, Distrito de Marracuene, Provincia de Maputo, Mozambique; Department of International Health, Johns Hopkins Bloomberg School of Public Health, 615 N. Wolfe Street, Baltimore, MD 21205, United States

**Keywords:** mobile phone survey, maternal health services, interactive voice response, computer-assisted telephone interviews, population-based statistics, survey methodology

## Abstract

The Coronavirus Disease 2019 (COVID-19) pandemic raised the need for rapid approaches for measuring population-based health indicators to understand its impact on service utilization, while avoiding face-to-face interviews. The Rapid Mortality Mobile Phone Surveys (RaMMPS) aimed to test the use of mobile phone surveys (MPS) for measuring maternal health services coverage in Mozambique by comparing estimates to the nationally representative Demographic Health Survey 2022 (DHS 2022). RaMMPS used two strategies: (i) subsampling from the existing national sample registration system, Country-wide Mortality Surveillance for Action (COMSA); (ii) Random Digit Dialing (RDD) of randomly generated phone numbers. We collected data from March to August 2022 for the COMSA MPS sample, and from June to December 2022 for the RDD sample. In both cases, computer-assisted telephone interviews were conducted among women aged 15–49 years. Women were asked questions on utilization of maternal health care (MHC) services for births within the 2 years preceding the survey. We compared adjusted MHC coverage estimates between each RaMMPS sample and the DHS 2022. Despite adjustment to redress the MPS samples, MHC coverage was higher in MPS samples compared to the DHS 2022 sample. The coverage of at least one antenatal care visit was 99% [95% confidence interval (CI): 98–99] in COMSA MPS and 98% (95% CI: 95–99) in RDD samples compared to 81% (95% CI: 79–84) in the DHS 2022 sample. Additionally, coverage of at least four antenatal care visits was 76% (95% CI: 73–80) in COMSA MPS and 85% (95% CI: 81–88) in RDD samples, compared to 48% (95% CI: 45–51) in the DHS 2022. Coverage of health facility delivery was 87% (95% CI: 84–90) in COMSA MPS and 90% (95% CI: 85–93) in RDD samples compared to 40% (95% CI: 38–62) in the DHS 2022. Similarly, skilled birth attendant coverage was 86% (95% CI: 82–89) and 88% (95% CI: 84–92), respectively, in COMSA MPS and RDD samples compared to 48% (95% CI: 46–49) in the DHS 2022. MHC coverage measured through MPS was consistently overestimated compared to nationally representative surveys, even after adjustments.

## Introduction

The COVID-19 pandemic caused country-wide shutdowns and suspended national face-to-face (FTF) survey data collection all over the world including the Mozambique Demographic Health Survey (DHS) which was paused during the planning stages in 2020 before resuming active field work in 2022. The COVID-19 pandemic highlighted the urgent need for rapid approaches for measuring population-based maternal, newborn, and child health (MNCH) and mortality statistics during a health crisis to understand its impact on MNCH services utilization, while avoiding FTF interviews. Although experience with mobile phone surveys (MPS) existed before the pandemic, a literature review of the MPS landscape by Gibson et al. in 2017 reported that few population-level MPS studies were conducted, and the majority of these were human operator or computer-assisted telephone interviews (CATI) using pre-existing sampling frames (Gibson et.al. 2017). However, since the pandemic, interest in MPS has increased substantially due to their ease in rapidly accessing a wide proportion of the population (Gourlay et al. 2021, Lambrecht et al. 2023), catalyzed by an unprecedented expansion in mobile phone networks and ownership in low- and middle-income countries (Greenleaf et al. 2020a). A recent study by Greenleaf et al. (2025) reported that 47% of young adults owned a mobile phone in Mozambique, with new mobile telephone subscriptions expected to increase in the next 5 years among the youth in Sub-Saharan Africa. These new youthful subscribers will help shape how mobile phones are used since they will be more likely to have smartphones, use the internet, and have social media (Greenleaf et al. 2025). The 2024 Mozambique DHS found that 45% of women and 66% of men aged 15–49 reported owning a mobile phone (Instituto Nacional de Estatística (INE) e ICF 2024). Additionally, MPS are less costly than traditional national health surveys (Gourlay et al. 2021, Vecino-Ortiz et al. 2021) and offer protection and anonymity for both survey interviewers and participants by avoiding FTF interviews and direct contact during national emergencies (Vecino-Ortiz et al. 2021) (Morgan et al. 2025). However, two questions remain: (i) Can MPS collect nationally representative samples? (ii) Can they be used to measure the coverage of maternal and child health interventions in a similar way as FTF surveys such as the DHS?

At the start of the COVID-19 pandemic in 2020, the World Bank implemented and launched high-frequency phone surveys (HFPS) in over 100 countries aimed at assessing the socio-economic impact the pandemic had on households. The HFPS aimed to provide real-time data that are representative at the national level, thereby allowing policy makers to make decisions based on the generated evidence and data (Hangoma et al. 2022, World Bank Group 2022). However, as with other MPS, results from the HFPS showed the selection biases that other MPS have encountered due to the pro-rich distribution of access/ownership of phones in the population, uneven cellular coverage, low response rates compared to traditional FTF surveys (Brubaker et al. 2021).

As part of an effort to assess whether MPS can collect nationally representative data and measure coverage and mortality indicators in a comparable way to FTF surveys, the Rapid Mortality Mobile Phone Surveys (RaMMPS) was developed and implemented in 2022 in four African countries and Bangladesh (Kante et al. 2025). The aim was to assess the feasibility and reliability of using mobile interviews to measure and monitor intervention coverage and child and adult mortality. The Mozambique RaMMPS included the testing of measures of childhood mortality using pregnancy history modules (Demographic Health Survey 2023) in a national sample of women of reproductive age. Questions were also included in the surveys to measure the coverage of maternal health care services. We analyze these questions to assess the accuracy of the coverage measures by comparing them to a national DHS survey in Mozambique. This study will help fill in the knowledge gap on whether population level estimates for antenatal care (ANC), facility delivery, and skilled attendant at birth coverages can be measured through MPS by comparing two different MPS methodologies to a standardized national survey such as the DHS.

## Materials and methods

### RaMMPS study design

The RaMMPS study in Mozambique was a cross-sectional mobile phone survey that implemented two approaches to reach women between 15 and 49 years of age. The first approach selected a sub-sample of phone numbers collected as part of an on-going mortality surveillance in the country called Country-wide Mortality Surveillance for Action (COMSA). COMSA is a nationally representative surveillance system that conducts routine surveillance of pregnancies, birth outcomes (pregnancy loss, stillbirths, births), and deaths in Mozambique (Macicame et al. 2023). The MPS sample drawn from the COMSA sample was based on a province-stratified random sample of households with available phone numbers. A total of 48 271 households were sampled across provinces with the expectation of reaching successfully 15 000 women (Kante et al. 2025). The second approach was random digit dialing (RDD) using a constructed mobile telephone numbers based on phone number structure in Mozambique. The implementation of the RDD approach involved a four-step process. First the RaMMPS team generated a large number of random phone numbers using STATA statistical software, based on the phone number structure in Mozambique and covered all three mobile phone companies in the country. Second, the phone numbers were screened using interactive voice survey (IVR), which is an automated phone system that calls potential participants and prompts callers to indicate their language preference and their interest in participating in the study using their telephone keypad. Additionally, the IVR approach was designed to account for multiple languages in Mozambique. The IVR implementation used a third-party web-based platform that allowed calling a large number of phones numbers simultaneously (EngageSpark 2025). Third, phone numbers that were eligible for the study, where the IVR respondent indicated their language preference, were called for interviews. A fourth step has been to monitor quotas by age group, urban/rural residence, and province of residence to improve the national spread and representativeness of the sample. The quota determination was based on the 2017 population census (Kante et al. 2025).

A survey questionnaire with a full pregnancy history module was administered to women in the COMSA MPS sample. The RDD sample was randomized into receiving a full pregnancy history module or a truncated pregnancy history module. As detailed previously by Kante et al., the randomization compared the full and truncated histories for accuracy in generating mortality estimates. The randomization resulted in equal samples during data collection for both the full and truncated pregnancy arms (Kante et al. 2025).

### Demographic Health Survey

The DHS is a nationally representative cross-sectional household survey conducted approximately every 5 or more years that interviews head of households and women of reproductive age (15–49 years). In Mozambique, the latest DHS was implemented in 2022–23 (DHS 2022) and included a module on pregnancy history collected from all women 15–49 years. Women who reported a pregnancy within the 2 years preceding the survey were asked a series of questions including (a) number of antenatal care visits, (b) timing of the first visit, (c) antenatal care by type of provider and antenatal care from a skilled provider, (d) antenatal care by place of delivery, (e) birth attendant. RaMMPS pregnancy history questionnaires were based on DHS modules, and women in both the RaMMPS COMSA and RDD samples were asked about their ANC and delivery care, as well as health services utilization in all pregnancies preceding the survey.

Both the COMSA and RDD approaches used CATI to collect the survey data. The COMSA MPS sample included 13 235 and the RDD/IVR sample 10 116 women of reproductive age 15–49 (Balanza et al. 2025). Due to constraints in reaching eligible minors via MPS, only emancipated minors were interviewed for RaMMPS as they were able to directly give verbal consent per our ethical approval. The survey based on the COMSA subsample was conducted from 9 March 2022 to 12 August 2022, while the RDD/IVR sample was surveyed from 23 June 2022 to 19 December 2022. Verbal informed consent was obtained from all participants via telephone.

### Data analysis

We compared the sociodemographic characteristics of the women interviewed, including age group, education level, marital status, province, and area of residence between the COMSA MPS and the RDD samples, and then compared them with the DHS 2022 sample. Weighted point estimates were generated for each sample for four maternal health care indicators: (1) at least one ANC visit (ANC1), (2) at least four ANC visits (ANC4), (3) birth delivery at a health facility, (4) birth attended by a skilled birth attendant. To account for potential bias introduced by sampling mobile phone owners and for non-response survey error (Holt and Smith 1979), a post-stratification method was used to redress the sample. The 2017 Mozambique Population Census ((INE) INdE 2019) data were used to derive the distribution of 15–49-year-old women by age, education, province, and place of residence, as well as household size, and a post-adjustment raking approach was applied to adjust the final sampling weights (Deville et al. 1993) using the Stata package “svycal” (Valliant and Dever 2018). The description of the construction of the final sampling weighting method is presented elsewhere (Kante et al. 2025). The analysis was conducted using Stata17 (StataCorp, College Station, Texas)

### Ethical approval

Ethical approval was obtained from the Mozambique National Institute of Health ethical review board (Our Ref: 93 CIBS-INS/2021) and the Johns Hopkins Bloomberg School of Public Health’s institutional review board (JHU IRB: 00016261).

## Results

### Characteristics of study respondents

Table 1 presents the distribution of women respondents aged 15–49 in the two surveys by socio-demographic characteristics, comparing the weighted and the unweighted proportions to the similar weighted proportions from the 2022 DHS. The unweighted distributions of women respondents in the COMSA MPS sample, were generally older (about 60% were between 30 and 49 years old), largely represented in urban areas (62%), and in Maputo City (24%), Maputo Province (18%), or Inhambane (11%). They were largely educated (about 90% had primary or more education level and 52% had secondary or more education level) and were married or living together (70%). The sample of women in the RDD presents a similar distribution, except that they were slightly younger than the COMSA MPS sample (42% were 30–49 years old). The unweighted mean age of women was 33 and 29 years, respectively, in the COMSA MPS and RDD samples compared to 28 years in DHS sample. This unweighted distribution is unlike the weighted nationally representative sample of women aged 15–49 years from the 2022 DHS, which shows a sample of women that are younger (39% were 30–49 years old), and residing in predominantly populous provinces (Nampula, 23%; Zambezia, 17%; Maputo Province, 10%; and Tete, 10%). Additionally, the 2022 DHS women sample resided largely in rural areas (61%) with higher proportion with no schooling (27%) and were married or living together (64%). Redressing the COMSA MPS or RDD samples using post-adjustment weighting aligned them with the DHS sample based on the selected demographic characteristics (Table 1).

**Table 1:** Unweighted and weighted demographic characteristics of women 15–49 years of age in the RaMMPS COMSA MPS, RaMMPS RDD, and DHS 2022 Mozambique surveys.

	Unweighted			Weighted		
	COMSA MPA	RDD	DHS 2022	COMSA MPS	RDD	DHS 2022
*N* (%)	13 235 (100)	10 116 (100)	13 183 (100)	13 235 (100)	10 116 (100)	13 183 (100)
Age group—*n* (%)						
15–19	685 (5.2)	1157 (11.4)	3109 (23.6)	3033 (22.9)	2295 (22.7)	3050 (23.1)
20–29	4651 (35.1)	4676 (46.2)	4695 (35.6)	5012 (37.9)	3806 (37.6)	4888 (37.1)
30–39	4254 (32.1)	2802 (27.7)	3121 (23.7)	3089 (23.3)	2362 (23.3)	3063 (23.2)
40–49	3645 (27.5)	1481 (14.6)	2258 (17.1)	2159 (16.3)	1653 (16.3)	2182 (16.6)
Province—*n* (%)						
Maputo Cidade	3129 (23.6)	1752 (17.3)	1259 (9.6)	581 (4.4)	442 (4.4)	655 (5.0)
Maputo Provincia	2330 (17.6)	2185 (21.6)	1276 (9.7)	1165 (8.8)	885 (8.7)	1347 (10.2)
Inhambane	1460 (11.0)	880 (8.7)	1008 (7.6)	709 (5.4)	542 (5.4)	555 (4.2)
Sofala	1192 (9.0)	970 (9.6)	1218 (9.2)	1111 (8.4)	838 (8.3)	909 (6.9)
Gaza	1007 (7.6)	748 (7.4)	1209 (9.2)	678 (5.1)	517 (5.1)	670 (5.1)
Nampula	605 (4.6)	934 (9.2)	1446 (11.0)	2623 (19.8)	1977 (19.5)	3064 (23.2)
Manica	837 (6.3)	640 (6.3)	1196 (9.1)	927 (7.0)	708 (7.0)	909 (6.9)
Zambezia	549 (4.1)	788 (7.8)	976 (7.4)	2387 (18.0)	1810 (17.9)	2193 (16.6)
Tete	699 (5.3)	548 (5.4)	1168 (8.9)	1250 (9.4)	962 (9.5)	1314 (10.0)
Cabo Delgado	820 (6.2)	402 (4.0)	1314 (10.0)	1049 (7.9)	797 (7.9)	705 (5.3)
Niassa	607 (4.6)	269 (2.7)	1113 (8.4)	813 (6.1)	639 (6.3)	861 (6.5)
Residence type—*n* (%)						
Urban	8167 (61.7)	8479 (83.8)	5695 (43.2)	5098 (38.5)	3880 (38.4)	5120 (38.8)
Rural	5068 (38.3)	1637 (16.2)	7488 (56.8)	8194 (61.9)	6236 (61.6)	8063 (61.2)
Education—*n* (%)						
None	1420 (10.7)	374 (3.7)	3033 (23.0)	2985 (22.6)	2260 (22.3)	3522 (26.7)
Primary	4891 (37.0)	2399 (23.7)	2426 (18.4)	6616 (50.0)	5031 (49.7)	5601 (42.5)
Secondary	6052 (45.7)	6188 (61.2)	4259 (32.3)	3462 (26.2)	2637 (26.1)	3709 (28.1)
Higher/other	872 (6.6)	1151 (11.4)	465 (3.5)	230 (1.7)	175 (1.7)	352 (2.7)
Marital status—*n* (%)						
Cohabitating/married	9142 (69.1)	6320 (62.5)	8195 (62.2)	9916 (74.9)	6860 (67.8)	8488 (64.4)
Never been married/single	2274 (17.2)	2806 (27.7)	3135 (23.8)	2122 (16.0)	2306 (22.8)	2896 (22.0)
Separated	1049 (7.9)	714 (7.1)	1145 (8.7)	696 (5.3)	611 (6.0)	1022 (7.8)
Widowed	647 (4.9)	220 (2.2)	422 (3.2)	461 (3.5)	244 (2.4)	378 (2.9)
Divorced	123 (0.9)	52 (0.5)	286 (2.2)	96 (.7)	81 (.8)	400 (3.0)

### Mobile phone survey performance

The survey response rates were 39% and 36%, respectively, for the COMSA MPS and the RDD samples (Noor et al. 2025). The refusal rate was similar between 8% in the COMSA MPS sample and 7% in the RDD sample. However, the COMSA MPS sample recorded a lower no response rate at 15%, where the mobile call was never answered compared to the RDD sample (26%). Conversely, the COMSA MPS sample had a higher percentage of numbers not accessible at 35%, compared to 27% in the RDD sample. Specifically, for the RDD sample, the IVR screened out about 90% of phone numbers which were not registered, not in use, or unresponsive.

### Coverage of maternal health care

**Figure 1:**
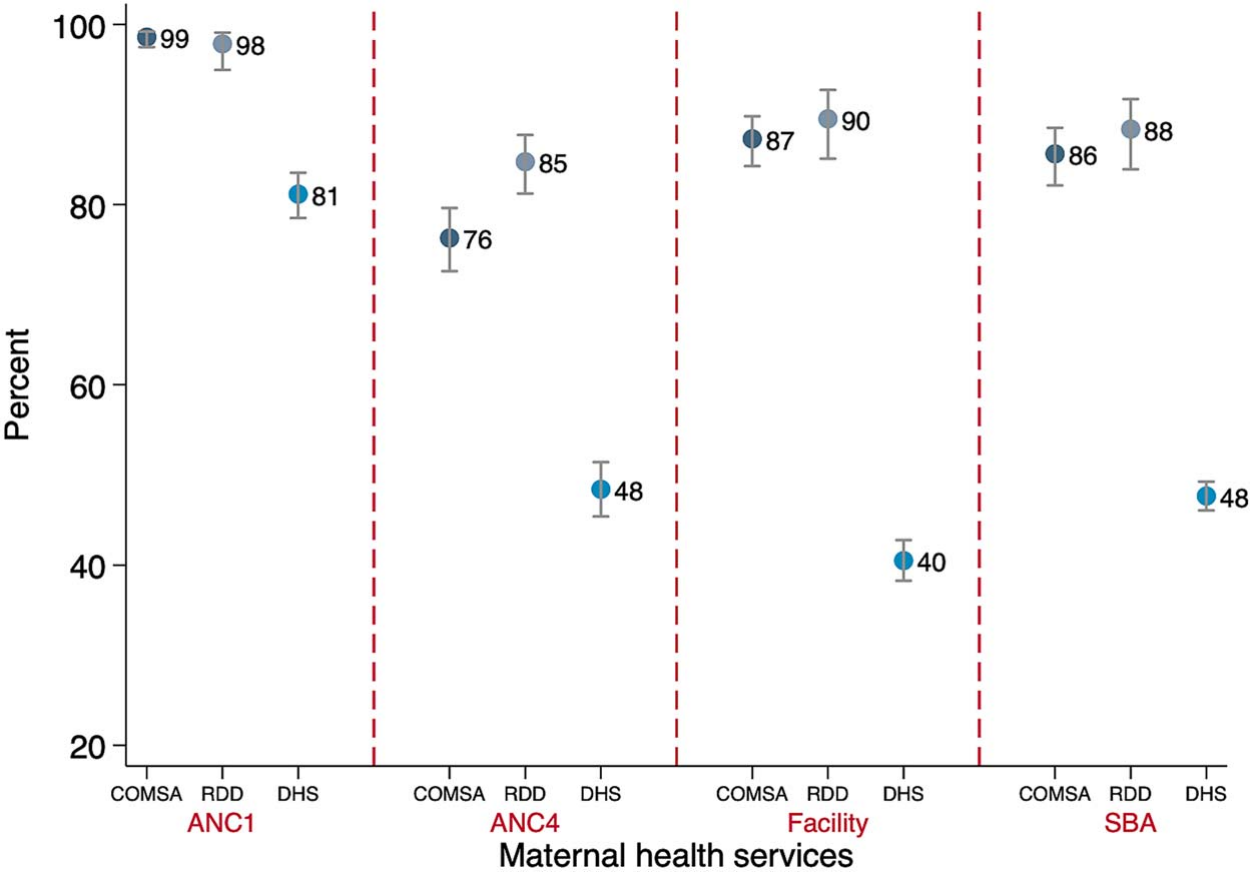
Weighted national level estimates comparing ANC1, ANC4, facility delivery, and SBA across COMSA, RDD, and DHS 2022.

Adjusted national coverage estimates for ANC1, ANC4, facility delivery, and SBA were significantly higher among the COMSA MPS and RDD samples compared to the DHS, considering that COMSA and RDD 95% confidence intervals (CIs) do not overlap with the DHS estimates (Fig. 1). For antenatal care coverage, 99% (95% CI: 98–99) COMSA and 98% (95% CI: 95–99) RDD samples reported receiving at least one antenatal care service during their pregnancy, compared to 81% (95% CI: 79–84) in the DHS. Similarly, ANC4 coverage was 76% (95% CI: 73–80) in the COMSA MPS sample, 85% (95% CI: 81–88) in the RDD sample, compared to 48% (95% CI: 45–51) in the 2022 DHS sample.

For intrapartum care coverage, facility delivery was 87% (95% CI: 84–90) in the COMSA MPS sample, 90% (95% CI: 85–93) in the RDD sample, compared to 40% (95% CI: 38–62) in the 2022 DHS sample. Similarly, SBA was 86% (95% CI: 82–89) in the COMSA MPS sample, 88% (95% CI: 84–92) in the RDD sample compared to 48% (95% CI: 46–49) in the 2022 DHS sample. The COMSA MPS and RDD samples produced similar coverage of ANC1, facility delivery, and SBA but the RDD sample generated higher coverage of ANC4 than the COMSA MPS sample (Fig. 1).

Table 2 presents the weighted coverage of ANC4 by women’s age group, place of residence, education, and marital status. ANC1 was close to universal (>95%); therefore, subgroup analysis was not presented. Consistently across all groups defined by these variables, coverage of ANC4 from the two RaMMPS samples was overestimated compared to the DHS estimates. The weighted coverage of ANC4 increased with age in both RaMMPS samples, ranging from 71% (95% CI: 61–79) among 15–19 years old to 81% (95% CI: 68–90) among 40–49 years old in the COMSA MPS sample, and from 85% (95% CI: 73–92) to 91% (95% CI: 81–96), respectively, in the RDD sample. However, the age-related gaps in ANC4 coverage were narrowed in the RaMMPS samples compared to the DHS. Similarly, the difference in the coverage of ANC4 between urban and rural areas in both RaMMPS samples was not as large as in the DHS sample, which showed higher coverage in urban compared to rural residence. In all three samples, coverage of ANC4 increased with maternal education level. However, the gap between women with no schooling and those with higher or other levels of education was wider in the DHS sample (50 percentage points difference) than in the COMSA sample (25 percentage points) and the RDD/IVR sample (8 percentage points). ANC4 coverage also varied slightly across marital status in all three samples.

**Table 2:** Weighted point estimates stratified by age group, residence type, education, and marital status for at least four antenatal care (ANC4) among women 15–49 years of age with a still or live birth 2 years preceding the survey in the RaMMPS COMSA MPS, RaMMPS RDD, and DHS 2022 Mozambique surveys.

*Weighted*	ANC4					
	COMSA MPS		RDD		DHS 2022	
	***n***	**% (95% CI)**	***n***	**% (95% CI)**	***n***	**% (95% CI)**
**Age group**						
15–19	816	71.0 (60.5–79.1)	640	84.7 (73.0–91.9)	367	45.9 (41.1–50.8)
20–29	1774	76.1 (71.5–80.2)	1252	82.8 (78.0–86.7)	1396	48.5 (45.1–52.0)
30–39	727	82.5 (78.2–86.1)	625	87.0 (80.6–91.6)	715	51.4 (47.5–55.3)
40–49	144	81.4 (67.5–90.3)	187	91.3 (80.5–96.4)	157	41.7 (34.2–49.7)
**Residence type—*n* (%)**						
Urban	1137	80.1 (72.9–85.8)	1041	85.7 (82.6–88.3)	1067	67.5 (63.8–70.9)
Rural	2323	74.5 (70.1–78.5)	1663	84.2 (78.6–88.5)	1566	40.6 (37.3–44.0)
**Education—*n* (%)**						
None	712	71.1 (62.1–78.7)	546	88.0 (78.2–93.8)	535	33.2 (29.2–37.4)
Primary	1880	77.2 (71.5–81.9)	1402	82.0 (75.8–86.9)	1261	48.0 (44.6–51.3)
Secondary	826	78.3 (70.9–84.3)	718	87.4 (83.9–90.3)	778	69.1 (65.5–72.4)
Higher/other	42	96.2 (91.2–98.4)	38	95.8 (92.1–97.8)	59	83.6 (70.6–91.5)
**Marital status—*n* (%)**						
Cohabitating/married	3092	77.1 (73.4–80.4)	2163	84.6 (80.6–87.9)	2190	53.7 (50.1–57.2)
Never been married/single	222	70.1 (50.8–84.2)	312	85.4 (67.9–94.1)	157	48.1 (41.1–55.1)
Separated	105	70.3 (53.9–82.8)	144	83.6 (62.1–94.0)	181	48.3 (41.8–55.0)
Widowed	25	76.3 (46.2–92.4)	25	75.0 (40.8–92.9)	41	58.0 (43.7–71.0)
Divorced	17	60.9 (18.8–91.3)	60	97.2 (68.2–99.8)	64	38.9 (28.8–50.1)

Additionally, Tables 3 and 4 present the weighted coverage of facility delivery and skilled attendant at birth by the same socio-demographic variables. Similar to ANC4, the coverage of facility delivery and skilled attendant at birth was consistently overestimated compared to the DHS sample. For both indicators, there were no differences in the coverage estimates by age group in both RaMMPS samples, while the DHS data showed decreasing coverage by age group. Rural areas had lower coverage than urban areas, although the difference was not as strong in the RaMMPS samples. Additionally, coverage of facility delivery increased with education level, but the gap between women with no schooling and those with higher education was smaller in the RaMMPS samples (7 percentage in COMSA MPS and 24 percentage points in the RDD sample) than in the DHS (29 percentage points difference). This trend was also observed in the SBA indicator between the COMSA MPS sample (8 percentage point difference) compared to the DHS (17 percentage points difference). However, the RDD sample produced the highest coverage (24 percentage points difference) compared to the COMSA MPS and DHS samples. There was no coverage difference by marital status in both RaMMPS samples, while coverage was highest among single women for both indicators in the DHS.

**Table 3:** Weighted point estimates stratified by age group, residence type, education, and marital status for health facility delivery, among women 15–49 years of age with a still or live birth 2 years preceding the survey in the RaMMPS COMSA MPS, RaMMPS RDD, and DHS 2022 Mozambique surveys.

*Weighted*	Facility delivery					
	COMSA MPS		RDD		DHS 2022	
	***n***	**% (95% CI)**	***n***	**% (95% CI)**	***n***	**% (95% CI)**
**Age group**						
15–19	1052	88.8 (79.8–94.0)	715	94.1 (83.3–98.1)	584	56.2 (51.1–61.2)
20–29	2053	86.5 (82.4–89.7)	1410	89.4 (82.5–93.7)	2134	39.8 (37.3–42.4)
30–39	776	87.6 (83.4–90.8)	611	84.7 (72.5–92.1)	1049	39.3 (36.5–42.2)
40–49	157	87.8 (76.0–94.3)	186	90.6 (74.3–97.0)	228	28.7 (24.0–34.0)
**Residence type—*n* (%)**						
Urban	1349	94.1 (91.1–96.2)	1149	93.7 (91.3–95.4)	1530	54.1 (51.4–56.8)
Rural	2689	84.2 (80.2–87.6)	1772	87.0 (80.0–91.8)	2465	35.0 (32.5–37.7)
**Education—*n* (%)**						
None	944	91.3 (86.9–94.3)	492	75.8 (57.7–87.8)	948	31.9 (28.9–35.1)
Primary	2062	82.9 (78.5–86.6)	1580	90.5 (85.3–93.9)	1872	38.7 (36.1–41.3)
Secondary	988	93.2 (83.2–97.4)	810	97.8 (96.6–98.5)	1101	57.0 (54.2–59.8)
Higher/other	44	98.6 (95.4–99.6)	40	99.2 (97.7–99.7)	74	60.2 (51.5–68.4)
**Marital status**—***n* (%)**						
Cohabitating/married	3535	86.5 (83.2–89.3)	2305	87.7 (82.4–91.5)	3298	43.7 (41.0–46.5)
Never been married/single	310	93.5 (84.6–97.4)	356	97.0 (93.3–98.7)	261	53.4 (47.4–59.4)
Separated	138	92.8 (84.0–97.0)	169	97.1 (90.7–99.1)	288	38.2 (34.0–42.6)
Widowed	31	93.7 (74.2–98.7)	30	91.5 (56.4–98.9)	52	34.3 (25.2–44.6)
Divorced	26	93.8 (74.4–98.7)	62	100.0(.0–.0)	96	29.1 (22.2–37.1)

**Table 4:** Weighted point estimates stratified by age group, residence type, education, and marital status for skilled birth attendant (SBA) among women 15–49 years of age with a still or live birth 2 years preceding the survey in the RaMMPS COMSA MPS, RaMMPS RDD, and DHS 202.

*Weighted*	Skilled birth attendant (SBA)					
	COMSA MPS		RDD		DHS 2022	
	***n***	**% (95% CI)**	***n***	**% (95% CI)**	***n***	**% (95% CI)**
**Age group**						
15–19	1040	87.8 (79.0–93.2)	700	92.1 (82.3–96.7)	675	65.0 (60.6–69.1)
20–29	2007	84.5 (79.7–88.4)	1391	88.2 (81.4–92.7)	2516	46.9 (45.0–48.8)
30–39	766	86.4 (82.5–89.5)	611	84.7 (72.5–92.1)	1211	45.4 (43.2–47.5)
40–49	149	82.8 (69.6–91.0)	183	89.1 (73.5–96.0)	301	38.0 (34.1–42.0)
**Residence type—*n* (%)**						
Urban	1343	93.7 (90.6–95.8)	1133	92.3 (89.8–94.2)	1522	53.8 (51.6–56.1)
Rural	2619	82.0 (77.4–85.9)	1752	86.0 (78.9–91.0)	3181	45.2 (43.3–47.1)
**Education—*n* (%)**						
None	924	89.4 (84.6–92.8)	492	75.8 (57.7–87.8)	1226	41.3 (38.9–43.9)
Primary	2020	81.3 (75.7–85.8)	1552	88.9 (83.4–92.7)	2305	47.6 (45.7–49.5)
Secondary	974	91.9 (82.6–96.4)	800	96.6 (94.7–97.9)	1099	56.9 (54.3–59.5)
Higher/other	43	96.9 (91.0–99.0)	40	98.8 (96.6–99.6)	71	57.5 (49.0–65.6)
**Marital status—*n* (%)**						
Cohabitating/married	3461	84.7 (80.9–87.9)	2268	86.3 (81.0–90.2)	3885	49.1 (47.2–51.0)
Never been married/single	312	94.3 (86.2–97.8)	357	97.1 (93.3–98.8)	287	58.7 (52.6–64.5)
Separated	132	89.0 (75.4–95.5)	169	97.1 (90.7–99.1)	330	43.8 (39.9–47.7)
Widowed	31	93.7 (74.2–98.7)	30	91.3 (56.9–98.8)	58	38.5 (29.6–48.2)
Divorced	25	93.3 (74.2–98.5)	62	100.0(.0–.0)	143	43.1 (36.8–49.7)

## Discussion

This study tested the use of mobile phone interviews to collect representative data on the coverage of maternal health interventions. The findings suggest that, while mobile phone interviews may be an expedited and low-cost strategy for data collection, caution must be considered with measuring outcomes such as maternal health care. Not only did the mobile phone interview samples, whether drawn from an existing database of phone numbers or constructed randomly using an RDD approach, yield samples that are biased toward wealthier populations, they may also lead to an overestimate of coverage results, even after adjustments. We found that the distribution of women aged 15–49 years old that were successfully interviewed in RaMMPS was different from a nationally representative distribution of 15–49-year-old women in the DHS sample, requiring adjustment weights using the raking method. The mobile phone sample was generally older, predominantly in urban areas and largely urbanized provinces. These findings are similar to previous studies that conducted mobile telephone surveys in other countries (Greenleaf et al. 2020a, 2020b, Lambrecht et al. 2023, Ramesh et al. 2023), where the sample of respondents reached had higher education and predominately resided in urban areas. Our study is the first national comparison of a mobile phone sample and the DHS to measure maternal health care coverage indicators that used a similar tool as the DHS and highlights the bias selection of more educated and urbanized respondents when collecting maternal services utilization data through the MPS method.

Compared to the DHS, the raking adjustment approach was successful in redressing the RaMMPS sample distribution to a representative sample. However, the coverage of maternal health services such as ANC1, ANC4, facility delivery, and SBA was overestimated when compared to traditional FTF surveys such as the DHS. In this study, we found gaps in the coverage estimates between the mobile phone sample and the DHS in the order of 17–18 percentage points (pp) for ANC1, 28–37 pp for ANC4, 47–50 for facility delivery, and 38–40 for skilled attendant at birth. This overestimation remained across groups defined by maternal age group, place of residence, education, and marital status. In a study examining the association between mobile phone ownership and maternal health services utilization using the 2010, 2014/15, and 2019/20 Rwanda DHS data, Tariku et al. found that women with mobile phones were more likely to use the recommended maternal health services and ensure their children were fully immunized. While mobile phone ownership increased from 40% to 71% from 2010 to 2019/20, mobile phone ownership favored wealthier and more educated households. Furthermore, Tariku et al. found that mobile phone ownership accounted for 58% of the disparity in antenatal care utilization in Rwanda (Tariku et al. 2025). This is consistent with our findings as the RaMMPS respondents, with access to a mobile phone, appeared to follow this trend where women were more educated and used more recommended ANC4, facility delivery, and SBA when compared to the DHS results.

Although this study did not collect wealth data, a multi-country mobile telephone survey conducted in Benin, India, and Malawi to evaluate the impact of a deworming program showed that smaller and poorer households were underrepresented in the survey in all three countries (Ramesh et al. 2023). A study by Sanchez-Paez et al. that looked at the effect of measuring under-five mortality and fertility by mobile phone ownership using DHS data from 34 low- and middle-income countries found that total fertility rate (TFR) and mortality rates were underestimated if data were only based on women who owned or had access to mobile phones. Additionally, TFR estimates remained biased even after poststratification weights using age, education level, area of residence, wealth, and marital status were applied (Sanchez-Paez et al. 2023). Similarly, a study by Greenleaf et al. comparing indicators of contraceptive use between FTF and RDD surveys among women showed a higher estimation of contraceptive use among the RDD group compared to the FTF group, even after adjusting for age, area of residence, and education level. Furthermore, the RDD group was younger and had higher education level compared to the FTF group (Greenleaf et al. 2020a).

Women’s utilization of maternal health services during pregnancy is influenced by education level (Chicumbe and Martins 2022). A study assessing the gender digital divide using DHS surveys from 23 countries showed that mobile phone ownership among women was highest among highly educated women residing in urban areas (MacQuarrie et al. 2022). Additionally, mobile phone ownership was highest in the 20–29 and 30–39 age groups (MacQuarrie et al. 2022). Greenleaf et al reported 47% mobile phone ownership and access among 15–24-year-olds in Mozambique, with higher phone ownership and access among 20–24-year-olds (51%) compared to their younger 15–19-year-old contemporaries (49%). Additionally, only 43% of women owned or had access to a mobile phone, with higher ownership in urban areas (61%) and secondary education (68%) (Greenleaf et al. 2025). Consistently, our study showed higher reported maternal health use in these age groups compared to younger or older age groups. Although women with no education were more likely to be unfamiliar or uncomfortable with mobile phone technology (USAID 2016), the coverage of maternal health services among women in the RaMMPS samples that reported no education was similar to or higher than among those that reported higher education in the DHS sample, suggesting behavioral differences between women who either own or have access to mobile phones compared to those who do not (Sanchez-Paez et al. 2023).

Selection bias (i.e. who is reached via MPS) played a large role in the RaMMPS data collection, despite trying to address it by monitoring the data collection with sample quotas for province, age groups, and urban/rural. The COMSA MPS and RDD RaMMPS samples used two different sampling frames but captured the same biased respondent samples. As previously highlighted, mobile phone access and ownership were highest among those in urban areas and more educated, which did not allow the RaMMPS survey to reach two-thirds of the Mozambican population that still resided in largely rural and densely populated provinces (Kante et al. 2025). Additionally, there may be potential reporting or mode effects, where respondents may report information differently via telephone interviews versus face-to face interviews. A study by Roberts et al. reported a stronger social desirability bias, where reported responses were more socially desirable to improve a respondent’s image, among telephone respondents compared to FTF respondents (Roberts et al. 2006). A stronger social desirability bias in MPS may contribute to higher reported maternal health services utilization compared to FTF DHS estimates.

### Limitations

A limitation of this study was that potential respondents may have been wary of the study calls, as most were unfamiliar with MPS and the study did not conduct a pre-study launch awareness campaign, potentially leading to more rejections or non-response of the RaMMPS study calls. To mitigate this, the call center staff shared the INS IRB phone number and email for potential respondents to verify the study’s legitimacy. However, additional reasons for non-response to the survey calls are unknown. Nevertheless, response rates achieved in the RaMMPS were typical of mobile phone interviews. A recent literature review conducted by Greenleaf et al. that examined CATI interviews in LMICs reported that response or cooperation rates for MPS, and specifically RDD, ranged from 3% to 52% (Greenleaf et al. 2026). Further, the RaMMPS study asked women about their ANC and health service use within 2 years prior to the survey, which was at the height of the COVID-19 shutdowns and may have altered their access to health services and utilization. However, the DHS also faced similar limitations in recall bias where women may not be able to accurately account for all ANC visits 2 to 5 years preceding the survey, as well as timing during the COVID shutdown disruptions to services.

## Conclusion

We found in this study that the RaMMPS national MPS, both the COMSA and RDD samples, overestimated the coverage of maternal health services compared to traditional FTF surveys such as the DHS. Our study compared two mobile phone survey methods and found that both methods overestimated ANC1, ANC4, facility delivery, and SBA coverage when compared to the most recent Mozambique DHS 2022 survey. Findings from these surveys must be carefully interpreted with a thorough understanding of the potential biases. MPS for data collection of maternal health services presented in this study require obtaining a representative sample that is similar to those obtained in FTF surveys, such as the DHS before using them for policy and program decisions. Although this study did not measure wealth, previous studies have also shown that it influences the uptake of maternal health services (Chicumbe and Martins 2022, MacQuarrie et al. 2022), which should be considered for future improvement for MPS. Therefore, further methodological approaches are also needed for the successful adjustment of mobile phone samples to produce nationally representative estimates. Recommendations for future studies should consider capturing wealth in the MPS, increasing sample sizes to help reach underrepresented or hard to capture groups, such as those with no education or younger respondents.

## Data Availability

RaMMPS study data are available upon reasonable request from HK, AA, or MK on the RaMMPS survey results. Mozambique DHS 2022 survey data are available for download on The DHS Program website at https://dhsprogram.com/.
